# Women With Ovulatory Cycles Have Longer Sleep, but Phases of Their Menstrual Cycles Do Not Differ in Sleep Characteristics

**DOI:** 10.1002/ajhb.70247

**Published:** 2026-03-24

**Authors:** Aleksandra Wachowicz, Andrzej Galbarczyk, Urszula M. Marcinkowska, Sude Ozdemir, Magdalena Klimek, Anna Tubek‐Krokosz, Kinga Słojewska, Karolina Krzych‐Miłkowska, Magdalena Mijas, Monika Ścibor, Grazyna Jasienska

**Affiliations:** ^1^ Department of Environmental Health, Faculty of Health Sciences Jagiellonian University Medical College Krakow Poland; ^2^ Lise Meitner Research Group BirthRites ‐ Cultures of Reproduction Max Planck Institute for Evolutionary Anthropology Leipzig Germany; ^3^ Doctoral School of the University of the National Education Commission Krakow Poland; ^4^ Jagiellonian University Medical College, Doctoral School of Medical and Health Sciences Krakow Poland; ^5^ Department of Anthropology Baylor University Waco Texas USA

**Keywords:** cyclical changes, LH surge, ovulation, REM, sleep quality, women's health

## Abstract

**Objectives:**

Duration and quality of sleep are influenced by many factors, including hormonal changes. The aim of this study was to investigate the differences between the phases of the menstrual cycle in total sleep duration and sleep stage distribution, specifically the duration of rapid eye movement (REM) phase, light, and deep sleep states and compare sleep parameters between ovulatory and anovulatory cycles.

**Methods:**

The study involved 130 women aged 20–35 (mean = 26.2 years; SD = 4.14). Ovulation was detected using luteinizing hormone (LH) urine tests. Sleep data were collected using the Fitbit Alta HR trackers, which measured total sleep time and the duration of sleep stages. Sleep parameters were analyzed separately for each of the five phases: menstrual bleeding, follicular, periovulatory, luteal, and premenstrual using repeated measures ANOVA. Differences between ovulatory and anovulatory cycles were assessed using Student's *t*‐test.

**Results:**

Women with the ovulatory cycle slept longer and had longer REM phases compared to women without ovulation. No statistically significant differences were observed in total sleep duration or sleep stage distribution across five phases of the menstrual cycle among women with detected ovulation.

**Conclusion:**

The findings suggest that ovulatory status might be associated with differences in total sleep time and REM sleep duration, whereas sleep duration and sleep stage distribution across menstrual cycle phases remain relatively constant. These results suggest that the presence of ovulation, rather than phase‐specific changes during the cycle, may play a more important role in shaping sleep characteristics.

## 1 Introduction

Sleep is a fundamental biological function, essential for physical, mental, and cognitive health (Walker 2017). It is a cyclical process embedded in the diurnal rhythm, involving a temporary loss of consciousness and reduced responsiveness to external stimuli. Both the duration and the architecture of sleep are crucial for proper functioning (Buysse 2014; St‐Onge et al. 2016). Sleep consists of two main states: nonrapid eye movement (NREM) and rapid eye movement (REM). These stages differ in brain activity and function, playing roles in emotion regulation, memory consolidation, and physical restoration (Carskadon and Dement 2005; Spencer 2006). NREM is further divided into light and deep sleep. Light sleep marks the transition from wakefulness and helps filter sensory input, while deep sleep, dominated by slow delta waves, is the most restorative stage, supporting recovery and immune function. REM sleep, in turn, is critical for emotional processing and memory integration (Carskadon and Dement 2005; Spencer 2006).

Among the many factors that influence sleep, biological variables specific to human females are of particular importance. Hormonal fluctuations across the lifespan, including those during pregnancy, the postpartum period, and menopause, are strongly associated with reduced sleep quality and more frequent awakenings (Nowakowski et al. 2013). One of the most prominent recurring hormonal processes in women's lives is menstrual cycles, which occur from puberty until menopause (Mihm et al. 2011). Each cycle is divided into several consecutive phases: menstrual bleeding, follicular, periovulatory, luteal, and premenstrual (Wilson et al. 2025). Each phase is marked by characteristic hormonal shifts that influence physiology, physical activity, mood, and behavior, including sleep (Bartelmez 1957; Baker and Lee 2022; Ozdemir et al. 2026).

Beyond the phase‐to‐phase shifts, the occurrence of ovulation itself represents a critical variable in sleep architecture. The variations are largely attributed to estradiol levels, which tend to be lower in nonovulatory cycles (Hambridge et al. 2013). Estradiol plays a vital role in supporting the body's natural daily rhythm, facilitating the transition to rest and ensuring the maintenance of the REM phase. Consequently, low estradiol levels are associated with shorter, lower‐quality sleep, potentially initiating a “vicious cycle” where poor sleep further suppresses hormonal recovery (Copinschi and Caufriez 2021).

During the luteal phase, when progesterone levels are elevated, sleep onset may be delayed and nocturnal awakenings more frequent (Driver et al. 2008; Manber and Bootzin 1997). REM sleep is reduced in this phase compared to the follicular phase (Patkai et al. 1974), while the premenstrual phase can be associated with pronounced sleep disturbances, particularly in women with premenstrual syndrome (PMS) (Mauri et al. 1988, Jeon and Baek 2023). However, findings remain inconsistent, as other studies have not observed significant differences in sleep parameters across menstrual phases (Lee et al. 1990). Such discrepancies highlight that individual responses to cyclical hormonal changes can vary widely in terms of sleep duration, depth, and vulnerability to disturbances. A recent systematic review by Nexha et al. (2024) provides a potential physiological explanation for these inconsistencies, suggesting that fluctuations in reproductive hormones may influence sleep through alterations in melatonin secretion, core body temperature, and circadian timing (Nexha et al. 2024). It has been also shown that PMS, dysmenorrhoea, and cycle disorders are associated with poorer sleep quality, daytime sleepiness, difficulty staying asleep, and reduced sleep duration (Jeon and Baek 2023). This indicates that sleep plays a multidimensional role in reproductive health and that sleep disturbances may exacerbate luteal phase symptoms.

Given these contrasting results, the aim of the present study was to investigate how various phases of the menstrual cycle differ in the total sleep duration, as well as in the distribution of sleep stages, including light sleep, deep sleep, and REM sleep.

## 2 Methods

Healthy women aged 20–35 (*N* = 234) were recruited in the urban area of Krakow, Poland via social media advertisements and campaigns. Before qualification to the study, participants underwent a telephone interview to assess their eligibility to participate in the study, including questions about health status and health behaviors. The inclusion criteria were regular menstrual cycles (no more than 5 days of difference between the cycles), no use of hormonal contraception within the past 3 months, and not being pregnant or breastfeeding within the 6 months prior to participation. Women with diagnosed gynecological or hormonal disorders (verified by an interview conducted by a Medical Doctor, a member of the research team) were also excluded from the study.

Ovulation status was evaluated using self‐performed urine tests detecting luteinizing hormone (LH; sensitivity 10 mIU/ml, Horien Medical, Solec Kujawski, Poland), performed daily between days 9 and 18 of the cycle, between 10:00 and 20:00, at approximately the same time each day, until a positive result was obtained. The tests show if LH levels are high enough to be detected, but they do not measure the exact hormone amount. These tests, together with menstrual calendars, were used to divide the cycle into five phases: (i) menstrual bleeding, (ii) follicular (from the end of menstrual bleeding to 2 days before positive LH test), (iii) periovulatory (2 days before, the day of, and 1 day after the positive LH test), (iv) mid luteal (from the end of the periovulatory phase to 6 days before menstruation), and (v) premenstrual (5 days before the next menstruation until the next menstrual bleeding) (de Jonge 2003).

Fitbit Alta HR wristbands (Fitbit Inc.; San Francisco, CA, USA) were used to monitor total sleep time and time spent in REM, light, and deep sleep stages. The device tracks sleep using an optical heart rate sensor and a 3‐axis accelerometer (Liu et al. 2019; Cook et al. 2019).

Out of 234 women enrolled in the study, 35 women who withdrew early (before the monitoring began) were excluded. From the remaining group, 69 participants whose sleep data were incomplete (more than 75% of nights missing) were then removed. As many as 53 women did not report the positive LH test. Following this order of exclusion, a total of 77 women with complete datasets were included in the final analysis of ovulatory cycles. In the first step, we compared a group of women who had a positive LH test (*N* = 77) with a group of women who did not have ovulation detected by the LH test (*N* = 53) in terms of age, cycle length, total sleep time, and individual sleep phases: REM, light, and deep sleep using a Student's *t*‐test. In the second step, differences in sleep duration across individual phases of the menstrual cycle were analyzed using repeated‐measures ANOVA, including only women with detected ovulation. In the last step, ANOVA analyses were repeated, controlling for the age of the participants. The data were analyzed using JASP version 0.95.4 software.

The study was performed in accordance with the Declaration of Helsinki. All participants provided written, informed consent. The study protocol received a positive opinion from the Bioethics Committee of the Jagiellonian University (no. 1072.6120.47.2018).

## 3 Results

First, demographic characteristics and basic sleep parameters were compared between participants with positive LH test (LH+ *N* = 77) and without positive LH test (LH− *N* = 53). A significant difference in age was observed between groups, with participants in the LH+ group being older than those in the LH− group (*p* = 0.021). Menstrual cycle length did not differ significantly between groups (*p* = 0.080) (Table 1).

**TABLE 1:** Comparison of women with positive LH test (LH+) and without positive LH test (LH−).

	LH+ (*N* = 77)				LH− (*N* = 53)				*t*	*p*
	Mean	SD	Minimum	Maximum	Mean	SD	Minimum	Maximum		
Age (years)	26.97	4.24	21	36	25.15	5.60	20	35	−2.33	0.021
Cycle length (days)	28.45	3.59	22	36	29.83	5.31	21	49	1.76	0.080
Total sleep time (minutes)	411.23	33.35	322.84	482.42	374.97	39.86	290.04	481.23	−5.62	< 0.001
REM sleep (minutes)	91.61	14.65	55.9	122.58	83.50	13.57	54	113.65	−3.19	0.002
Light sleep (minutes)	224.89	29.09	178.78	330.46	243.24	32.66	177.14	337.25	−0.30	0.762
Deep sleep (minutes)	74.73	11.17	51.38	95.5	71.47	10.99	44.45	104.13	−1.65	0.102

Abbreviation: REM, rapid eye movement.

A significant difference was found in total sleep length and REM sleep duration, which were longer among LH+ participants compared with those without detected ovulation (*p* < 0.001 and *p* = 0.002, respectively). No significant differences between groups were observed for light sleep duration (*p* = 0.762) or deep sleep duration (*p* = 0.102). Detailed characteristics of sleep stages in different menstrual phases and results of Student's *t*‐test are presented in Table 1.

The final study group consisted of women with positive LH test with an average age of 27 years (SD = 4.21). Among them, ovulation occurred, on average, on day 14 of the cycle (SD = 2.64), but only 11.69% of participants ovulated on day 14. The average length of the menstrual cycle was 28 days (SD = 3.59). Cycle length ranged from 22 to 42 days (Table 1).

**FIGURE 1:**
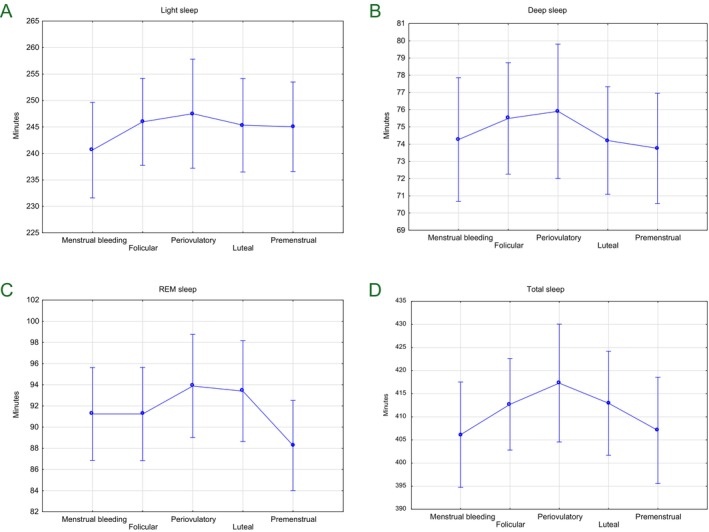
Mean duration of sleep stages light (A), and deep sleep (B), REM (C) and total sleep time (D) across different phases of the menstrual cycle on figures. None of the differences were statistically significant.

There were no statistically significant differences in sleep characteristics among phases of menstrual cycle (Figure 1). Controlling for age of participants did not change the results. Detailed values for sleep stages in different menstrual phases and results of a repeated measures ANOVA are presented in Table 2.

**TABLE 2:** Mean duration of total sleep and sleep stages (REM, light, and deep sleep) across different phases of the menstrual cycle among women with positive LH test (*N* = 77).

Phases of sleep	Phases of the menstrual cycle					*F*(df)	*p*	*η* ^2^ₚ	*F*(df)^a^	*p* ^a^	*η* ^2^ *ₚ* ^a^
	Menstrual bleeding	Follicular	Periovulatory	Luteal	Premenstrual						
Light sleep (minutes)	240.63	245.97	247.51	245.33	245.04	0.56 (4, 304)	0.690	0.01	0.95 (4,300)	0.495	0.01
Deep sleep (minutes)	74.27	75.49	75.91	74.21	73.76	0.51 (4, 304)	0.737	0.01	0.05 (4,300)	0.954	< 0.01
REM phase sleep (minutes)	91.24	91.24	93.88	93.4	88.27	1.64 (4, 304)	0.165	0.02	1.05 (4, 300)	0.387	0.01
Total sleep time (minutes)	406.14	412.70	417.30	412.94	407.06	0.93 (4, 304)	0.446	0.01	1.13 (4,300)	0.346	0.02

Abbreviation: REM, rapid eye movement.

^a^ Adjusted for age.

## 4 Discussion

The aim of this study was to examine whether sleep duration and sleep stage distribution vary across menstrual cycle phases in healthy, regularly menstruating young women. Although trends were observed, for example, slightly shorter REM sleep in the premenstrual phase or longer deep sleep around ovulation, none of observed differences reached statistical significance.

Previous research has indicated that hormonal fluctuations across the menstrual cycle can influence sleep patterns. During the luteal phase, when progesterone levels are elevated, increased sleep difficulties such as longer sleep onset latency, more frequent awakenings, and reduced REM sleep were reported (Driver et al. 2008; Manber and Bootzin 1997; Patkai et al. 1974). This phase has also been linked to poorer sleep quality, particularly among women experiencing PMS (Mauri et al. 1988; Baker and Lee 2022). However, in our study, accelerometer‐based data did not reveal significant sleep quality shifts across menstrual phases among young, healthy women. This discrepancy might result from differences in measurement methods, given that earlier findings were based primarily on self‐reported data (Driver, Manber, and Mauri), and objective recordings from accelerometers may capture sleep differently.

Further, a likely explanation for this discrepancy is the considerable individual variability in how women respond to hormonal changes. While some women experience marked sleep disruptions across their cycle, others show no perceptible changes (Lee et al. 1990). This variability may be influenced by psychological sensitivity to hormonal shifts, genetic predispositions, or preexisting sleep characteristics (Nowakowski et al. 2013), but these mechanisms remain insufficiently understood and under‐researched.

Another possible source of incongruent results is methodological approaches adopted by the up‐to‐date studies. Many earlier studies relied on estimated cycle phases based on calendar dates alone, sometimes solely the date of the onset of the menstrual cycle, without physiological confirmation of either the end of the cycle or the ovulation date (Bartelmez 1957; Patkai et al. 1974; Mauri et al. 1988; Lee et al. 1990). This can lead to misclassification which may result in biased conclusions. In contrast, our study used LH testing to detect ovulation and define menstrual cycle phases. Without such physiological verification, claims about phase‐specific sleep changes should be interpreted with caution. Moreover, the history of exploration of this topic may have skewed our understanding of the studied phenomenon. Studies reporting null results such as ours may have remained unpublished, leading to an overrepresentation of positive findings in the literature (Baker and Lee 2022; Lee et al. 1990).

Interestingly, we have found differences between women who ovulated as detected by the LH test and women without positive LH test in terms of average total sleep length and average REM phase length. This may be due to differences in estradiol levels, as estradiol might be lower in nonovulatory cycles compared to ovulatory cycles (Hambridge et al. 2013). Estradiol supports the body's natural daily rhythm, helping it recognize the right time for rest and ensuring the REM phase lasts long enough. When levels are low, sleep becomes shorter and poorer in quality, which further decreases estradiol and creates a “vicious cycle” that hinders recovery (Copinschi and Caufriez 2021). However, it is important to note that a negative LH test does not provide definitive confirmation that ovulation did not occur (Mihm et al. 2011).

The use of home‐based LH surge testing for ovulation detection and phase differentiation carries inherent limitations. Other methods, such as measuring the mid‐cycle drop in estradiol or using ultrasound, can improve accuracy but are time‐consuming, costly, and difficult to use in larger studies (Mihm et al. 2011; Marcinkowska et al. 2018). In addition, this study did not include direct hormone measurements or information about mood symptoms, such as PMS. This is an important limitation because hormone levels vary widely across the menstrual cycle, and stronger PMS symptoms have been linked to changes in sleep duration and sleep quality (Lee et al. 1990; Mauri et al. 1988; Baker and Lee 2022). The lack of these data may have influenced the sleep results. Future studies should include hormonal measures and assessments of mood symptoms to better understand how menstrual cycle changes affect sleep.

The missing data in this study may not be random. It could be hypothesized that women with higher intra‐individual variability across the menstrual cycle, for instance those who suffered from intensified symptoms during the luteal and premenstrual phases such as sleep disturbances, physical discomfort, or mood changes may have a lower tolerance for wearing the wrist‐worn device. This could lead to selective noncompliance during periods of increased symptomatic burden. Such patterns of missingness are a recognized challenge in wearable‐based research, where physiological or psychological distress can directly impact a participant's willingness to maintain continuous monitoring (Simblett et al. 2018).

Finally, the use of Fitbit devices in our study, while user‐friendly, comes with limitations. Although validated for general sleep tracking (Liu et al. 2019; Cook et al. 2019), the device has reduced accuracy in detecting specific sleep stages (de Zambotti et al. 2018) and overestimates wakefulness and sleep fragmentation (Haghayegh et al. 2019). Specifically, validation studies against polysomnography have shown that while these devices are accurate in tracking REM sleep, they tend to overestimate light sleep and underestimate deep sleep (de Zambotti et al. 2018). More sensitive measures such as polysomnography would be needed to confirm subtle changes in sleep architecture. Additionally, our study did not account for variables such as sleep onset latency, number of awakenings, or subjective sleep quality, all of which may be more responsive to hormonal fluctuations than just sleep duration.

This study provides additional insight into how menstrual phases may be related to real‐life sleep patterns outside the laboratory. The results indicate that in healthy, young women, sleep appears relatively stable across the cycle, although individual variability is highly possible. Levels of sex hormones produced during menstrual cycles show high intra‐individual variation, even among women with regular cycles (Jasienska et al. 2017). These observations highlight the need for future research using more sensitive measurement tools, controlling for external factors (Baker and Lee 2022; Liu et al. 2019), and moving beyond calendar‐based estimation of menstrual phases. Future studies should also include women with PMS or other menstrual‐related disorders to explore whether they are more vulnerable to cycle‐related sleep changes.

## 5 Conclusion

In this study, statistically significant differences were found between women with ovulatory and anovulatory cycles, with women in ovulatory cycles demonstrating longer total sleep time as well as longer REM sleep duration compared to those without detected ovulation. There was no statistically significant differences detected in total sleep time or in the duration of REM, light, and deep sleep duration across the phases of the menstrual cycle in ovulating women. These findings suggest that for healthy women sleep structure may remain relatively stable throughout the menstrual cycle. However, due to individual differences and the possible limitations of wearable sleep trackers, further research using larger samples and more precise tools (e.g., polysomnography) is recommended. Understanding the relationship between the menstrual cycle and sleep can be helpful in identifying and managing sleep disturbances in women.

## Author Contributions

**Aleksandra Wachowicz:** writing, data verification, data analysis, literature search. **Andrzej Galbarczyk:** writing, reviewing and editing text, data analysis, literature search. **Urszula M. Marcinkowska:** editing and reviewing text, data analysis, literature search. **Sude Ozdemir:** data verification, editing text. **Magdalena Klimek:** data collector, editing and reviewing text. **Anna Tubek‐Krokosz:** data collector, editing and reviewing text. **Kinga Słojewska:** data collector, editing and reviewing text. **Karolina Krzych‐Miłkowska:** data collector, editing and reviewing text. **Magdalena Mijas:** data collector, editing and reviewing text. **Monika Ścibor:** editing and reviewing text. **Grazyna Jasienska:** project leader, editing and reviewing text.

## Funding

This study was supported by the National Science Centre (2017/25/B/NZ7/01509) and the Salus Publica Foundation.

## Conflicts of Interest

The authors declare no conflicts of interest.

## Data Availability

The data that support the findings of this study are available on request from the corresponding author. The data are not publicly available due to privacy or ethical restrictions.

## References

[ajhb70247-bib-0001] Baker, F. C. , and K. A. Lee . 2022. “Menstrual Cycle Effects on Sleep.” Sleep Medicine Clinics 17, no. 2: 283–294. 35659080 10.1016/j.jsmc.2022.02.004

[ajhb70247-bib-0002] Bartelmez, G. W. 1957. “The Phases of the Menstrual Cycle and Their Interpretation in Terms of the Pregnancy Cycle.” American Journal of Obstetrics and Gynecology 74, no. 5: 931–955. 13469878 10.1016/0002-9378(57)90137-0

[ajhb70247-bib-0003] Buysse, D. J. 2014. “Sleep Health: Can We Define It? Does It Matter?” Sleep 37, no. 1: 9–17. 24470692 10.5665/sleep.3298 PMC3902880

[ajhb70247-bib-0004] Carskadon, M. A. , and W. C. Dement . 2005. “Normal Human Sleep: An Overview.” Principles and Practice of Sleep Medicine 4, no. 1: 13–23.

[ajhb70247-bib-0029] Cook, J. D. , S. C. Eftekari , E. Dallmann , M. Sippy , and D. T. Plante . 2019. “Ability of the Fitbit Alta HR to Quantify and Classify Sleep in Patients with Suspected Central Disorders of Hypersomnolence: A Comparison Against Polysomnography.” Journal of Sleep Research 28, no. 4: e12789. 30407680 10.1111/jsr.12789

[ajhb70247-bib-0006] Copinschi, G. , and A. Caufriez . 2021. “Sleep and the Ovarian Axis.” Current Opinion in Endocrine and Metabolic Research 17: 38–45. 10.1016/j.coemr.2021.01.001.

[ajhb70247-bib-0007] de Jonge, X. A. J. 2003. “Effects of the Menstrual Cycle on Exercise Performance.” Sports Medicine 33: 833–851. 12959622 10.2165/00007256-200333110-00004

[ajhb70247-bib-0008] de Zambotti, M. , A. Goldstone , S. Claudatos , I. M. Colrain , and F. C. Baker . 2018. “A Validation Study of Fitbit Charge 2 Compared With Polysomnography in Adults.” Chronobiology International 35, no. 11: 1468–1476. 10.1080/07420528.2017.1413578 29235907

[ajhb70247-bib-0009] Driver, H. S. , F. C. Baker , D. Mitchell , and I. M. Colrain . 2008. “The Menstrual Cycle Effects on Sleep.” Sleep Medicine Clinics 3, no. 1: 1–11.

[ajhb70247-bib-0010] Haghayegh, S. , S. Khoshnevis , M. H. Smolensky , K. R. Diller , and R. J. Castriotta . 2019. “Accuracy of Wristband Fitbit Models in Assessing Sleep: Systematic Review and Meta‐Analysis.” Journal of Medical Internet Research 21, no. 11: e16273. 31778122 10.2196/16273 PMC6908975

[ajhb70247-bib-0011] Hambridge, H. L. , S. L. Mumford , D. R. Mattison , et al. 2013. “The Influence of Sporadic Anovulation on Hormone Levels in Ovulatory Cycles.” Human Reproduction 28, no. 6: 1687–1694. 23589536 10.1093/humrep/det090 PMC3657125

[ajhb70247-bib-0013] Jasienska, G. , R. G. Bribiescas , A.‐S. Furberg , S. Helle , and A. Núñez‐de la Mora . 2017. “Human Reproduction and Health: An Evolutionary Perspective.” Lancet 390, no. 10093: 510–520. 28792413 10.1016/S0140-6736(17)30573-1

[ajhb70247-bib-0030] Jeon, B. , and J. Baek . 2023. “Menstrual Disturbances and its Association with Sleep Disturbances: A Systematic Review.” BMC Women's Health 23, no. 1: 470. 10.1186/s12905-023-02629-0. 37658359 PMC10474748

[ajhb70247-bib-0014] Lee, K. A. , J. F. Shaver , E. C. Giblin , and N. F. Woods . 1990. “Sleep Patterns Related to Menstrual Cycle Phase and Premenstrual Affective Symptoms.” Sleep 13, no. 5: 403–409. 2287852

[ajhb70247-bib-0015] Liu, J. , W. T. Wong , I. M. Zwetsloot , Y. C. Hsu , and K. L. Tsui . 2019. “Preliminary Agreement on Tracking Sleep Between a Wrist‐Worn Device Fitbit Alta and Consensus Sleep Diary.” Telemedicine and e‐Health 25, no. 12: 1189–1197. 30601109 10.1089/tmj.2018.0202

[ajhb70247-bib-0016] Manber, R. , and R. R. Bootzin . 1997. “Sleep and the Menstrual Cycle.” Health Psychology 16, no. 3: 209–214. 9152698 10.1037//0278-6133.16.3.209

[ajhb70247-bib-0017] Marcinkowska, U. M. , A. Galbarczyk , and G. Jasienska . 2018. “La Donna è Mobile? Lack of Cyclical Shifts in Facial Symmetry, and Facial and Body Masculinity Preferences – A Hormone Based Study.” Psychoneuroendocrinology 88: 47–53. 29161637 10.1016/j.psyneuen.2017.11.007

[ajhb70247-bib-0018] Mauri, M. , R. L. Reid , and A. W. MacLean . 1988. “Sleep in the Premenstrual Phase: A Self‐Report Study of PMS Patients and Normal Controls.” Acta Psychiatrica Scandinavica 78: 82–86. 3177000 10.1111/j.1600-0447.1988.tb06304.x

[ajhb70247-bib-0019] Mihm, M. , S. Gangooly , and S. Muttukrishna . 2011. “The Normal Menstrual Cycle in Women.” Animal Reproduction Science 124, no. 3–4: 229–236. 20869180 10.1016/j.anireprosci.2010.08.030

[ajhb70247-bib-0020] Nexha, A. , L. Caropreso , T. de Azevedo Cardoso , J. S. Suh , A. C. Tonon , and B. N. Frey . 2024. “Biological Rhythms in Premenstrual Syndrome and Premenstrual Dysphoric Disorder: A Systematic Review.” BMC Women's Health 24, no. 1: 551. 10.1186/s12905-024-03395-3. 39375682 PMC11457342

[ajhb70247-bib-0021] Nowakowski, S. , J. Meers , and E. Heimbach . 2013. “Sleep and Women's Health.” Sleep Medicine Research 4, no. 1: 1–22. 25688329 10.17241/smr.2013.4.1.1 PMC4327930

[ajhb70247-bib-0022] Ozdemir, S. , A. Galbarczyk , A. Wachowicz , et al. 2026. “Variations in Physical Activity Across the Menstrual Cycle in Healthy Women: A Focus on Step Count and Activity Intensity.” American Journal of Human Biology 38, no. 2: e70216. 41703916 10.1002/ajhb.70216

[ajhb70247-bib-0023] Patkai, P. , G. Johannson , and B. Post . 1974. “Mood, Alertness and Sympathetic‐Adrenal Medullary Activity During the Menstrual Cycle.” Psychosomatic Medicine 36: 503–512. 4473792 10.1097/00006842-197411000-00005

[ajhb70247-bib-0024] Simblett, S. , B. Greer , F. Matcham , et al. 2018. “Barriers to and Facilitators of Engagement With Remote Measurement Technology for Managing Health: Systematic Review and Content Analysis of Findings.” Journal of Medical Internet Research 20, no. 7: e10481. 10.2196/10480 PMC6062692 30001997

[ajhb70247-bib-0025] Spencer, A. 2006. Psychologia Współczesna. Gdańskie Wydawnictwo Psychologiczne.

[ajhb70247-bib-0026] St‐Onge, M. P. , M. A. Grandner , D. Brown , et al. 2016. “Sleep Duration and Quality: Impact on Lifestyle Behaviors and Cardiometabolic Health: A Scientific Statement From the American Heart Association.” Circulation 134, no. 18: e367–e386. 27647451 10.1161/CIR.0000000000000444 PMC5567876

[ajhb70247-bib-0027] Walker, M. 2017. Why We Sleep: Unlocking the Power of Sleep and Dreams. Scribner.

[ajhb70247-bib-0028] Wilson, M. , K. Lee , D. E. Ehrlich , et al. 2025. “Cycle Effects Are Not Universal: A Case Study of Urinary C‐Reactive Protein.” Biology 37, no. 1: e24207. 10.1002/ajhb.24207 PMC11724170 39794910

